# Developing cellulolytic *Yarrowia lipolytica* as a platform for the production of valuable products in consolidated bioprocessing of cellulose

**DOI:** 10.1186/s13068-018-1144-6

**Published:** 2018-05-15

**Authors:** Zhong-peng Guo, Julien Robin, Sophie Duquesne, Michael Joseph O’Donohue, Alain Marty, Florence Bordes

**Affiliations:** LISBP, CNRS, INSA/INRA UMR 792, Université de Toulouse, 135, Avenue de Rangueil, 31077 Toulouse, France

**Keywords:** *Yarrowia lipolytica*, Cellulolytic biocatalyst, Consolidated bioprocessing, Cellulose, Lipids, Recombinant protein production, Ricinoleic acid

## Abstract

**Background:**

Both industrial biotechnology and the use of cellulosic biomass as feedstock for the manufacture of various commercial goods are prominent features of the bioeconomy. In previous work, with the aim of developing a consolidated bioprocess for cellulose bioconversion, we conferred cellulolytic activity of *Yarrowia lipolytica*, one of the most widely studied “nonconventional” oleaginous yeast species. However, further engineering this strain often leads to the loss of previously introduced heterologous genes due to the presence of multiple *LoxP* sites when using *Cre*-recombinase to remove previously employed selection markers.

**Results:**

In the present study, we first optimized the strategy of expression of multiple cellulases and rescued selection makers to obtain an auxotrophic cellulolytic *Y. lipolytica* strain. Then we pursued the quest, exemplifying how this cellulolytic *Y. lipolytica* strain can be used as a CBP platform for the production of target products. Our results reveal that overexpression of *SCD1* gene, encoding stearoyl-CoA desaturase, and *DGA1*, encoding acyl-CoA:diacylglycerol acyltransferase, confers the obese phenotype to the cellulolytic *Y. lipolytica*. When grown in batch conditions and minimal medium, the resulting strain consumed 12 g/L cellulose and accumulated 14% (dry cell weight) lipids. Further enhancement of lipid production was achieved either by the addition of glucose or by enhancing cellulose consumption using a commercial cellulase cocktail. Regarding the latter option, although the addition of external cellulases is contrary to the concept of CBP, the amount of commercial cocktail used remained 50% lower than that used in a conventional process (i.e., without internalized production of cellulases). The introduction of the *LIP2* gene into cellulolytic *Y. lipolytica* led to the production of a strain capable of producing lipase 2 while growing on cellulose. Remarkably, when the strain was grown on glucose, the expression of six cellulases did not alter the level of lipase production. When grown in batch conditions on cellulose, the engineered strain consumed 16 g/L cellulose and produced 9.0 U/mL lipase over a 96-h period. The lipase yield was 562 U lipase/g cellulose, which represents 60% of that obtained on glucose. Finally, expression of the hydroxylase from *Claviceps purpurea* (CpFAH12) in cellulolytic *Y. lipolytica* procured a strain that can produce ricinoleic acid (RA). Using this strain in batch cultures revealed that the consumption of 11 g/L cellulose sustained the production of 2.2 g/L RA in the decane phase, 69% of what was obtained on glucose.

**Conclusions:**

In summary, this study has further demonstrated the potential of cellulolytic *Y. lipolytica* as a microbial platform for the bioconversion of cellulose into target products. Its ability to be used in consolidated process designs has been exemplified and clues revealing how cellulose consumption can be further enhanced using commercial cellulolytic cocktails are provided.

**Electronic supplementary material:**

The online version of this article (10.1186/s13068-018-1144-6) contains supplementary material, which is available to authorized users.

## Background

Key elements of the bioeconomy are the use of renewable biomass as raw material and industrial biotechnology as an enabling technology for advanced manufacturing. Accordingly, for decades research worldwide has focused on the use of lignocellulosic biomass as a feedstock for fermentation processes aimed at the production of a wide variety of commercial products, including fuels, chemicals and enzymes [1, 2]. Despite enormous efforts and considerable R&D expenditure, the use of lignocellulosic biomass (LCB) as a raw material for manufacturing has still not reached maturity, mainly because it is difficult to extract fermentable sugars from this composite matter in an economically viable way.

The three major components of LCB are cellulose, hemicelluloses and lignin. Cellulose accounts for 40–60% of LCB dry matter content and its extraction and conversion into glucose constitute the key initial steps in current LCB biorefinery concepts [3]. The basic macromolecular components of cellulose are linear β-1,4-glucan chains, with glucose being the only molecular constituent of these. Despite the fact that cellulose is chemically homogeneous, in plant cells it displays a high degree of structural complexity, because the β-1,4-glucan chains are tightly packed into microfibrils surrounded by hemicelluloses and embedded in a lignin matrix [4, 5]. Accordingly, for biorefinery strategies employing enzyme-based approaches, biomass must first be pretreated to break down the lignocellulose matrix, release cellulose microfibrils and increase the frequency of amorphous zones [6]. Although many different pretreatment strategies have been proposed, those belonging to the organosolv technology family are reputed for their ability to solubilize lignin and produce quite pure amorphous cellulose that is amenable to enzyme action [7, 8].

Once cellulose is obtained in a form suitable for enzyme hydrolysis, a variety of enzymes are required to produce glucose. In typical commercial cocktails based on the secretome of *Trichoderma* sp., chain cleaving endoglucanases (EGs) are associated with cellobiohydrolases (CBHs) that act at chain extremities and remove cellodextrins in a progressive fashion, and β-glucosidases (BGLs) that hydrolyze these latter, producing glucose [9]. Although the hydrolytic efficiency of cellulase cocktails has been greatly improved in recent years [4], hydrolysis of cellulose is still regarded as the major cost driver [6]. Therefore, the reduction of enzyme costs in biorefining is still a key aim for research. In this respect, consolidated bioprocessing (CBP) represents a paradigm shift, since this strategy involves the internalization of enzyme production using a cellulolytic organism that is able to hydrolyze cellulose and convert glucose into a target product in a single integrated process step. Accordingly, the economic burden of enzyme production is drastically reduced and further gains are achieved through the reduction of capital costs [10]. Nevertheless, the main challenge in establishing CBP is the identification of a suitable bifunctional microorganism that displays both potent cellulolytic activity and the ability to produce target molecules in a commercially relevant manner (e.g., high yield and titer). Unfortunately, this is not an easy task, since very few naturally occurring microorganisms simultaneously satisfy these requirements [6].

Attempts so far to obtain process-ready CBP microorganisms have not yet provided a satisfactory solution [1]. In cases where cellulolytic activity has been conferred to microorganisms using strain engineering, low cellulose hydrolysis rates remain a problem. This is partly due to difficulties linked to the overexpression of multiple cellulase components (i.e., EGs, CBHs and BGLs). However, it is also due to failure to properly match the cellulase mixture to the target cellulose pulps, adjusting the ratio of the different components to suit the specific composition of the substrate [11].

**Fig. 1:**
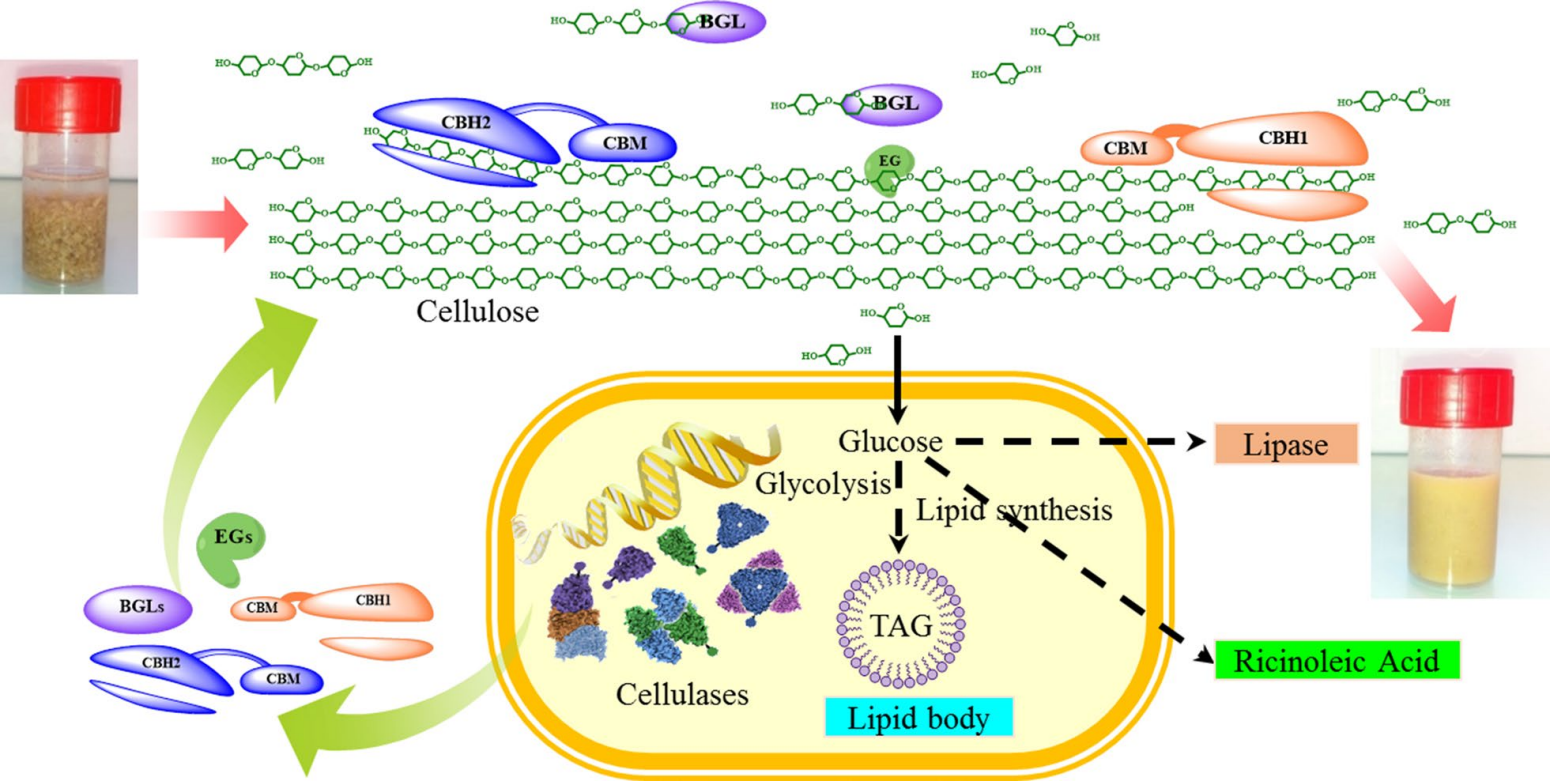
Strategies used in the current study to develop a cellulolytic *Y. lipolytica* for CBP of cellulose. Products of interest are shown in color boxes

As a potential candidate for CBP, the yeast *Yarrowia lipolytica* is interesting because it has been already used by industry for different applications and displays the ability to accumulate lipids, which are potential biofuel precursors [12–14]. Moreover, *Y. lipolytica* performs a wide range of post-translational modifications, secretes enzymes at high levels and is amenable to genetic manipulation [15]. Exploiting these attributes, we have recently developed a cellulolytic *Y. lipolytica* strain producing BGLs, EGs and CBHs, tailoring the relative proportions of these so that they optimally hydrolyze an Organosolv cellulose pulp [16]. To further engineer this strain, it is necessary to use *Cre*-recombinase to remove previously employed selection markers. However, this operation is complicated, because it often leads to the loss of previously introduced heterologous genes due to the presence of multiple *LoxP* sites [17, 18]. Therefore, in this work, while further optimizing the expression of multiple cellulases, we have rescued selection makers to obtain an auxotrophic cellulolytic *Y. lipolytica* strain. Subsequently, we have further engineered the strains for the production of three different target products, namely lipase, lipids and ricinoleic acid. Accordingly, we have demonstrated how this microbial platform can be used for CBP, leading to the production of commercially valuable products (Fig. 1).

## Methods

### Strains and media

The genotypes of the microbial strains used in the present study are summarized in Table 1. *E. coli* DH5 were purchased from Invitrogen (Paisley, UK) and used for plasmid construction. Restriction enzymes and DNA polymerase were purchased from New England Biolabs (Evry, France). Unless otherwise stated, all reagents were purchased from Sigma (Saint Quentin-Fallavier, France). *Y. lipolytica* strains were routinely cultivated in a medium composed of 10 g/L yeast extract, 10 g/L Bacto Peptone and 10 g/L glucose (YPD). Transformants were selected on solid YNB medium (1.7 g/L YNB, 10 g/L glucose or cellobiose, 5 g/L ammonium chloride, 50 mM sodium–potassium phosphate buffer at pH 6.8, and with (for Ura^+^) or without (for Leu^+^) 2 g/L casamino acids, supplemented with uracil (440 mg/L), leucine (440 mg/L) or hygromycin (200 μg/mL) depending on the selection marker requirements. Solid media contained 1.5% agar. The detection of endoglucanase activity in solid YNBcasa medium was achieved by incorporating 2 g/L Azo-CM-Cellulose (Megazyme). For lipid production in bioreactors, yeasts were first grown on minimal medium (MM) containing vitamins, trace elements [19] and salts, including 7.5 g/L (NH_4_)_2_SO_4_, 7.5 g/L K_2_HPO_4_, 7.5 g/L NaH_2_PO_4_ and 1.0 g/L MgSO_4_·7H_2_O with 50 g/L glucose, and then supplemented with 100 g/L Organosolv cellulose (cellulose content ≈ 91%), the latter being provided by CIMV S.A (Levallois-Perret, France). For lipid production in shake flasks, the minimal medium containing 80 g/L glucose or 72.8 g/L Organosolv cellulose, and 2.7 g/L (NH_4_)_2_SO_4_ (*C*/*N* ratio ≈ 30:1) was used. Lipase and ricinoleic acid were produced in YTD (10 g/L yeast extract, 20 g/L tryptone, 25 g/L glucose and 100 mM citrate buffer, pH 5.5) and YTC media (10 g/L yeast extract, 20 g/L tryptone, 27.5 g/L Organosolv cellulose and 100 mM citrate buffer, pH 5.5). YT media containing yeast extract, tryptone and citrate buffer (100 mM, pH 5.5) were used as control.

**Table 1:** Microbial strains used in the present study

Strain	Parental strain	Relevant genotype/expressed gene	Source of references
*E. coli* DH5		Φ80dlacZΔm15, *recA1*, *endA1*, *gyrA96*, *thi*-*1*, *hsdR17* (rk^−^, mk^+^), *supE44*, *relA1*, *deoR*, Δ(*lacZY*A-argF) U169	Invitrogen
YLp	Po1d	*MATA*, *leu2*-*270*, *ura3*-*302*, *xpr2*-*322*, *∆pox1*-*6*	[27]
YLpW	YLp	*LEU2*, *URA3*	[32]
YLpO	YLp	*pTEF*-*SCD1*-*LEU2*, *pEXP*-*DGA1*-*URA3*	This investigation
YLpL	YLp	*LEU2*, *pTEF*-*LIP2*-*URA3*	This investigation
YLx	YLp	Δ*DGAT1*-*2*, Δ*LRO1*, Δ*FAD2*	[22]
YLxW	YLx	*LEU2*, *URA3*	This investigation
YLxR	YLx	*LEU2*, *URA3*, *pTEF*-*CpFAH12*-*Hygro*^*R*^	This investigation
CYLp	YLp	*pEXP*-*BGL1*, *pHTEF*-*BGL*2, *pHTEF*-*EG1, pEXP*-*EG2*, *pHTEF*-*CBH1*, *pHTEF*-*CBH2*-*LEU2*	This investigation
CYLpW	CYLp	*URA3*	This investigation
CYLpO	CYLp	*pTEF*-*SCD1*, *pEXP*-*DGA1*-*URA3*	This investigation
CYLpL	CYLp	*pTEF*-*LIP2*-*URA3*	This investigation
CYLx	YLx	*pEXP*-*BGL1*, *pHTEF*-*BGL*2, *pHTEF*-*EG1*, *pEXP*-*EG2*, *pHTEF*-*NcCBH1*-*URA3*, *pHTEF*-*TrCBH2*-*LEU2*	This investigation
CYLxR	CYLx	*pTEF*-*CpFAH12*-*Hygro*^*R*^	This investigation

### Plasmid and strain construction

The plasmids constructed in the present study are summarized in Table 2 and primers are listed in Table 3. A schematic diagram of strain construction is shown in Additional file 1: Figure S1. The cellulolytic *Y. lipolytica* was constructed as described previously [16] with the following modifications. The TEF promoter present in plasmids JMP62UraTB1 and JMP62LeuTrEG2 (for the expression of *Y. lipolytica* BGL1 and *T. reesei* EG2, respectively) was replaced by the EXP1 promoter. This was achieved by fusing the EXP1 promoter, amplified from the genomic DNA of *Y. lipolytica* by PCR using primers F1/R1, with the PCR fragment amplified from the plasmid JMP62UraTB1 (primers F2/R2) and JMP62LeuTrEG2 (primers F3/R3), respectively, using In-Fusion^®^ HD Cloning Kit (Clontech, USA). Subsequently, the expression cassette *P*_*EXP1*_-*BGL1*, amplified from the vector JMP62UraEXP-BGL1 by PCR (primers F4/R4), was fused with the vector JMP62LeuTrEG1 linearized by *Cla*I, yielded BGL1/EG1 co-expressing plasmid JMP62LeuEB1TE1 (Additional file 1: Figure S2). Similarly, fusing the expression cassette *P*_*EXP1*_-*EG2*, amplified from the vector JMP62LeuEXP-EG2 by PCR (primers F4/R4), with the *Cla*I-linearized vector JMP62UraB2, yielded the plasmid JMP62UraTB2EE2 that directs co-expression of BGL2 and EG2 (Additional file 1: Figure S3). Afterward, vectors JMP62LeuEB1TE1, JMP62UraTB2EEII, JMP62UraHNcCBH1 (for expressing *N. crassa* CBH1) and JMP62LeuHTrCBH2 (for expressing *T. reesei* CBH2) were linearized by *Not*I and introduced into *Y. lipolytica* YLp and YLx strain using the lithium acetate method [20], resulting in strains CYLp and CYLx. Transformants were first screened on YNB plate based on auxotrophic genotype, and then tested for growth on cellobiose and for degradation of Azo-CMC. Clones displaying both activities were retained for further analysis. During strain construction, the *LoxP*-Cre recombination system was used for marker rescue and to ensure the multistep insertion of the target genes [17]. After each cycle of gene transformation, ten transformants of each construct were cultivated in liquid YNB media, and the transformants that showed the fastest growth rates and produced the highest level of cellulases were selected for further engineering.

**Table 2:** Plasmids used or constructed in the present study

Plasmids	Description	Source of references
JMP62UraTEF	*URA3*, *pTEF*	[33]
JMP62LeuTEF	*LEU2*, *pTEF*	[33]
PUB4-CRE	*hph, hp4d*-*CRE*	[17]
JMP62UraTrEG1	*URA3*, *pTEF*-*EG1*	[16]
JMP62LeuTrEG2	*LEU2*, *pTEF*-*EG2*	[16]
JMP62UraHNcCBH1	*URA3*, *pHTEF*-*NcCBH1*	[16]
JMP62LeuHTrCBH2	*LEU2*, *pHTEF*-*TrCBH2*	[16]
JMP62UraTB1	*URA3*, *pTEF*-*BGL1*	[32]
JMP62LeuB2	*LEU2*, *pTEF*-*BGL2*	[32]
JMP62UraEXP	*URA3*, *pEXP*	This investigation
JMP62LeuEB1TE1	*LEU2*, *pEXP*-*BGL1, pHTEF*-*EG1*	This investigation
JMP62UraTB2EE2	*URA3*, *pHTEF*-*BGL2, pEXP*-*EG2*	This investigation
JMP62UraED1	*URA3*, *pEXP*-*DGA1*	This investigation
JMP62UraTS1	*URA3*, *pTEF*-*SCD1*	This investigation
JMP62UraED1TS1	*LEU2*, *pEXP*-*DGA1, pTEF*-*SCD1*	This investigation
JMP62UraTEF-LIP2	*URA3*, *pTEF*-*LIP2*	[21]
JMP62UraTEF-*Cp*FAH12	*URA3*, *pTEF*-*FAH12*	[22]

**Table 3:** Sequences of oligonucleotide primers used in this study

Primer names	Sequence (5′-3′), 15-bp homologous sequence for infusion is underlined
F1	GAGTTTGGCGCCCGTTTTTTCG
R1	TGCTGTAGATATGTCTTGTGTGTAAGGGGG
F2	GACATATCTACAGCAGGATCCCACAATGATCTTCTCTCT
R2	ACGGGCGCCAAACTCATCGATTCTAGGGATAACAGGGTAA
F3	GACATATCTACAGCAGGATCCCACAATGAAGCTTTCC
R3	ACGGGCGCCAAACTCATCGATTCTAGGGATAACAGGGTAATTA
F4	TTATCCCTAGAATCGGAGTTTGGCGCCCGTTTTTTCG
R4	CTTGCGGCGGCATCGGAATTCGATTTGTCTTAGAGGAACGCATATACA
F5	CAGCAGGATCCCACAATGACTATCGACTCACAATACTACAAGTCG
R5	TGAGAACCCCCTAGGTTACTCAATCATTCGGAACTCTGGG
F6	CCGAAGGATCCCACAATGGTGAAAAACGTGGACCAAGTGG
R6	TGAGAACCCCCTAGGCTAAGCAGCCATGCCAGACATACCG
JMP1F	CCTAGGGGGTTCTCACCATCATCAC
JMP1R	TGTGGGATCCTGCTGTAGATATGTCTTG
JMP2F	CCTAGGGGGTTCTCACCATCATCACC
JMP2R	TGTGGGATCCTTCGGGTGTGAGTTG

To enhance lipid production, *DGA1 gene* and *SCD1* gene (GenBank accession codes XM_504700.1 and XM_501496.1, respectively) that encode acyl-CoA:diacylglycerol acyltransferase and stearoyl-CoA desaturase (catalyzes Δ9-desaturation of palmitoyl-CoA and stearoyl-CoA to palmitoleoyl-CoA and oleoyl-CoA), respectively, were PCR amplified from the genomic DNA of *Y. lipolytica* using primers F5/R5 and F6/R6, respectively. A 15-base pair sequence homologous to the target plasmid was introduced at the 3′ and 5′ ends of each gene during PCR amplification. Then, *DGA1* and *SCD1* genes were fused with the PCR fragment of vector JMP62UraEXP (primers JMP1F/JMP1R) and JMP62UraTEF (primers JMP2F/JMP2R), respectively. Afterward, the *P*_*EXP1*_-*DGA1* expression cassette was PCR amplified from the vector JMP62UraEXP-DGA1 using primers F4 and R4, and was fused with the *Cla*I-linearized vector JMP62UraTEF-SCD1 to yield JMP62UraED1TS1. This plasmid was transformed into *Y. lipolytica* YLp and CYLp strains, yielding YLpO and CYLpO, respectively.

For the expression of *Y. lipolytica* lipase 2, JMP62UraTEF-LIP2 [21] was transformed into YLp and CYLp strains, yielding YLpL and CYLpL, respectively.

For the production of ricinoleic acid, JMP62UraTEF-CpFAH12 containing the gene encoding *Claviceps purpurea* fatty acid Δ12-hydroxylase gene (*CpFAH*12) under the control of TEF promoter [22] was transformed into YLx and CYLx to yield strains YLxR and CYLxR, respectively.

After construction, all expression vectors were verified by DNA sequencing (GATC Biotech, Konstanz, Germany). The successful integration of multiple genes into the genome of *Y. lipolytica* was verified by PCR using gene-specific primers (Additional file 1: Table S1; Figure S4). After that, ten transformants of each construct were cultivated in liquid YNB media, and the transformant that showed the highest growth rate and produced the highest level of cellulases was selected for further analysis. Table 1 summarizes the expressed cellulase genes and their corresponding *Y. lipolytica* transformants.

### Lipid production in Erlenmeyer flasks

Yeasts were pre-cultivated in minimal media until stationary phase and then used to inoculate 100 mL minimal media containing 80 g/L glucose or 72.8 g/L Organosolv cellulose in 1 L Erlenmeyer flasks to yield an initial OD value of 1.0. Cellic^®^ CTec2 (Novozymes) was added at the enzyme loading of 10 or 20 FPU/g cellulose for the cultures containing Organosolv cellulose. Cultures were grown at 28 °C under shaking at 140 rpm for 5 days. Samples were taken at regular intervals to quantify glucose, biomass, lipids and residual cellulose.

### Lipid production in bioreactors

Yeasts were pre-cultivated in minimal medium until stationary phase and then used to inoculate 1.2 L minimal medium in 5 L stirred-tank BIO-STAT B-PLUS bioreactors (Sartorius, Frankfurt, Germany) to yield an initial OD value of 1.0. The pH was maintained at 5.5 by automatic addition of 2 M KOH. An aeration of 1.0 vvm was used and the stirring speed was set to 1200 rpm to ensure a dissolved oxygen tension of at least 20% of air saturation. The batch phase was designed to produce biomass and cellulases on glucose (50 g/L) under normal growth conditions (nitrogen was not limited). An initial Organosolv cellulose addition of 50 g/L was conducted when the concentration of glucose dropped around 20 g/L. Organosolv cellulose addition (50 g/L) together with vitamins and trace elements was carried out for a second time when the carbon dioxide concentration of off-gas dropped and remained unchanged as determined by the Prima PRO process mass spectrometer (Thermo Fisher Scientific, Cheshire, UK). In addition, NH_4_Cl was added to the medium to yield a C/N ratio of 30:1 for each cellulose feeding. For the control experiments, the fed-batch phase was started with exponential feeding of glucose at a concentration of 500 g/L into the bioreactors at a specific feed rate of 0.1 h^−1^ with the initial feed rate of 20 mL/h for 10 h. Then a constant feed rate of 20 mL/h was applied for 8 h, after which the concentration of glucose in bioreactors dropped below 20 g/L. In addition to vitamins and trace elements, NH_4_Cl was added to yield a C/N ratio at 30:1. Foaming was controlled automatically by the addition of TEGO Antifoam KS911 (Evonik Goldschmidt GmbH, Germany). Samples were taken regularly to quantify glucose, cellulose, lipids, extracellular protein and biomass, as well as cellulase activities. The results are shown as the mean value of three independent experiments.

### Lipase and ricinoleic acid production

Yeasts were pre-cultivated in YPD medium until stationary phase and then used to inoculate 100 mL YT, YTD or YTC in 1 L Erlenmeyer flasks to yield an initial OD value of 1.0. Cultures were grown at 28 °C under shaking at 140 rpm for 5 days. For ricinoleic acid production, decane was added into the medium at a ratio of 1–10 (v/v). Samples were taken at regular intervals to monitor enzyme activities and quantify biomass, residual cellulose and ricinoleic acid.

### Cellulase and lipase activity assay

Total cellulase activity was measured using the filter paper assay (FPU) following National Renewable Energy Laboratory (NREL) standard biomass analytical procedures [23].

Lipase activity in the culture supernatant was measured by monitoring the release of *p*-nitrophenol from *p*-nitrophenyl butyrate (pNPB) as described previously [21].

All protein concentrations were measured using the Bradford method and bovine serum albumin as a standard [24]. All enzymatic activity measurements were performed in triplicate unless otherwise stated.

### Analysis of glucose consumption and product formation

To determine the concentration of glucose, triplicate culture samples (1.5 mL each) were removed from growing cultures and rapidly frozen in liquid nitrogen, then thawed on ice before centrifugation (8000×*g* for 5 min at 4 °C) and recovery of supernatants. Glucose was measured using an Aminex HPX87-H column (Bio-Rad Laboratories, Germany), operating at 50 °C using a mobile phase (5 mM H_2_SO_4_) flowing at a rate of 0.5 mL/min. Glucose was detected using a Shodex RI-101 refractive index detector (Showa Denko, New York, NY).

Lipids were extracted from freeze-dried cells (~ 10 mg) and methylated as described previously [25]. During the lipid extraction, C17:0 (50 μg) was added as the internal standard and fatty acid methyl esters (FAMEs) were analyzed by gas chromatography (6890 N Network GC System, Agilent, USA). Fatty acids in the decane phase, including ricinoleic acid, were silylated by BSA (N,O-bis (trimethylsilyl) acetamide) before analysis. Briefly, 20 μL of samples from the decane phase were mixed with 180 µL decane and 5 μL of BSA. The mixture was incubated at room temperature for half an hour and then processed for GC analysis. Measurements were performed in split mode (1 μL at 250 °C), with helium as the carrier gas (2 mL/min). FAMEs were separated on an HP-5 GC column (30 m × 0.32 mm I.D., 0.5-μm film thickness, Agilent, USA). The temperature program was 120 °C, ramped up to 180 °C (10 °C/min) for 6 min, 183 °C (0.33 °C/min) for 9 min and 250 °C (15 °C/min) for 5 min. Detection was performed using a flame ionization detector (FID) at 270 °C (2.0 pA). FAMEs were quantified by comparing their profiles with that of standards of known concentrations.

### Analysis of residual cellulose and determination of dry cell weight

To determine the dry cell weight for cultures grown on glucose, triplicate samples (2 mL each) were removed and filtered using pre-weighed PES filters (0.45 μm; Sartorius Biolab, Germany). The biomass retained on the filters was washed, dried in a microwave oven at 150 W for 15 min and then placed in a desiccator before weighing.

For cultures grown on cellulose, the quantification of cellulose residues and dry cell matter was conducted as previously described with slight modifications [26]. Briefly, yeast cell biomass and residual cellulose were recovered from samples using centrifugation at 8000×*g* for 10 min at 4 °C. After supernatant removal, the pellet (*P*) was washed two times with distilled water, using centrifugation between each wash and the pellet was freeze dried and weighed. Afterward, pellets were treated independently with Cellic^®^ CTec2 and (1.0% w/v) dilute sulfuric acid at 121 °C for 1 h, and the amount of glucose released in each case was quantified using HPLC analysis as described above. Dry cell weight was deduced by subtracting the amount of cellulose from the weight of *P*. The biomass yield was calculated as the ratio of the amount of biomass obtained divided by the amount of carbon source consumed.

### Fluorescence microscopy

A Leica DM 4000B microscope was used to capture phase contrast and fluorescence images at 100× oil immersion magnification. Yeast cells were stained with BODIPY dye (0.1 μg/mL for 5 min). Samples were excited at 505 nm and fluorescence emission (around 515 nm) was collected for analysis.

## Results and discussion

### Construction of a cellulolytic *Y. lipolytica* as a platform for consolidated bioprocessing of cellulose to produce valuable products

In previous work, we have shown that efficient cellulolytic activity can be conferred to *Y. lipolytica* [16] by simultaneously expressing two BGLs, two EGs and two CBHs in specific ratios. To achieve this, relevant enzyme-encoding sequences under the control of strong promoters (e.g., TEF and HTEF containing multiple repeats of upstream activation sequence) were inserted into the *Y. lipolytica* genome. Each insertion was followed by the removal of selection markers using *Cre*-recombinase. Therefore, in the final construction we estimated that 8 *LoxP* sites remained on the genome (Additional file 1: Figure S5). However, we nevertheless observed definitive loss of one or several previously introduced heterologous genes during selection marker removal. This is probably caused by random recombination, which is due to the presence of multiple *LoxP* sites [18]. Therefore, to resolve this issue we set out to reduce the number of *LoxP* sites that are introduced during *Y. lipolytica* engineering. To achieve this, the different cellulolytic enzymes were introduced pairwise into the genome using extended gene cassettes containing two genes with different promoters (EXP and HTEF). The EXP promoter was chosen because it displays a strength similar to that of the TEF promoter (Additional file 1: Figure S6). Using this strategy, the number of *LoxP* sites introduced during the construction of cellulolytic *Y. lipolytica* was reduced by two (Additional file 1: Figure S5). More importantly, excision of the selection marker was achieved without causing the loss of previously integrated genes. Finally, cellulolytic ability was successfully conferred to two different *Y. lipolytica* host strains, YLp and its derivative YLx, yielding strains CYLp (cellulolytic YLp) and CYLx (cellulolytic YLx). Cultivation of these strains in flasks on both minimal (MM) and rich medium (YTD) revealed that the expression of the six cellulases led to a small decrease in maximum specific growth rates (*μ*_max_) and biomass yields, compared with the corresponding parental strains (Table 4).

**Table 4:** Comparison of growth and cellulase activity of recombinant *Y. lipolytica* strains grown on 25 g/L glucose in shake flasks under normal growth condition for 2 days

	YLpW	CYLpW	CYLpO	CYLpL	YLxW	CYLx	CYLxR
Minimal media							
*μ*_max_ (h^−1^)	0.16 ± 0.00	0.15 ± 0.01	0.15 ± 0.01	0.14 ± 0.01	0.11 ± 0.01	0.09 ± 0.00	0.08 ± 0.01
*Y*x/s (g DCW/g)	0.55 ± 0.01	0.51 ± 0.01	0.54 ± 0.01	0.49 ± 0.01	0.32 ± 0.01	0.26 ± 0.00	0.24 ± 0.01
Cellulase activity (FPU/mL)	n.d.	0.18 ± 0.02	0.18 ± 0.01	0.16 ± 0.01	n.d.	0.15 ± 0.02	0.13 ± 0.01
YTD							
*μ*_max_ (h^−1^)	0.43 ± 0.01	0.38 ± 0.01	0.37 ± 0.01	0.36 ± 0.01	0.16 ± 0.02	0.14 ± 0.01	0.12 ± 0.01
*Y*x/s (g DCW/g)	0.92 ± 0.06	0.83 ± 0.04	0.82 ± 0.05	0.80 ± 0.03	0.72 ± 0.03	0.65 ± 0.02	0.63 ± 0.03
Cellulase activity (FPU)	n.d.	0.84 ± 0.05	0.86 ± 0.03	0.82 ± 0.04	n.d.	0.70 ± 0.02	0.64 ± 0.03

*n.d.* not detectable

The results were calculated from at least three biological replicates and are given as the mean values ± standard deviation

### Lipid production by engineered *Y. lipolytica* strains in shake flasks

**Fig. 2:**
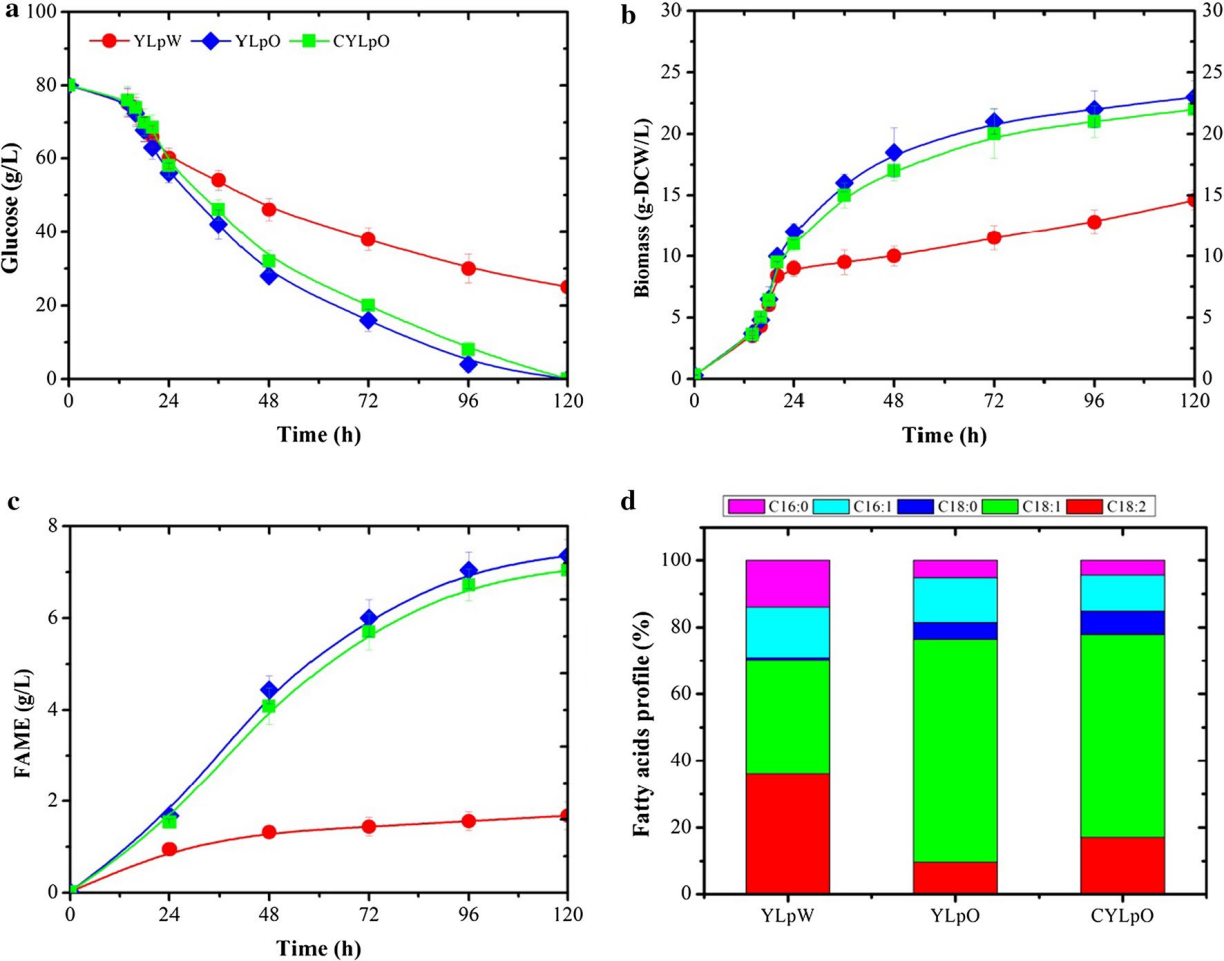
Comparison of lipid production by recombinant *Y. lipolytica* strains YLpW (prototrophic ∆pox strain), YLpO (∆pox strain overexpressing *SCD1* and *DGA1*) and CYLpO (cellulolytic ∆pox strain overexpressing *SCD1* and *DGA1*) during aerobic batch culture in minimum media supplemented with 80 g/L glucose under nitrogen starvation. Shown are **a** glucose consumption, **b** biomass formation and **c** FAME production versus time, and **d** the fatty acids profile. Values plotted at each time point are the means of three replications. Error bars represent standard deviation from the means

Previous work has shown that the deletion of the 6 *POX* genes (i.e., creation of *Y. lipolytica* YLp strain) that encode the peroxisomal acyl-coenzyme oxidases involved in lipid β-oxidation leads to increased lipid accumulation [27]. Additionally, it has been shown that the simultaneous overexpression of SCD1, DGA1 and acetyl-CoA carboxylase (ACC1) of *Y. lipolytica* yields an obese phenotype [28, 29]. Therefore, to investigate the use of CYLp as a CBP platform for cellulose to biofuel process, lipid biosynthesis was enhanced in CYLp by overexpressing SCD1 and DGA1, yielding strain CYLpO. Overexpression of the lipid biosynthesis pathway in CYLpO did not influence either the growth or enzyme production, irrespective of the culture medium used (Table 4). Moreover, batch cultivation of CYLpO in shake flasks and comparison with YLpW and YLpO (a YLp strain overexpressing SCD1 and DGA1) confirmed that the overexpression of SCD1 leads to faster glucose consumption and biomass production in lipid accumulation phase under nitrogen starvation (Fig. 2). Likewise, the use of fluorescence microscopy to observe intracellular lipids revealed that both YLpO and CYLpO displayed the obese phenotype (Additional file 1: Figure S6). However, contrary to previous reports [29], overexpression of SCD1 did not procure a significant increase in the exponential growth rate in this work. This is likely due to the use of different culture conditions and the fact that, unlike in previous work, the *ACC1* gene was not overexpressed, because of limitations imposed by the use of selection markers. Consequently, YLpO and CYLpO produced 7.3 g/L FAME (30% of DCW) and 7.0 g/L FAME (29% of DCW), respectively, which is higher than FAME production by YLpW (1.7 g/L FAME, 12% of DCW) but less than the previously reported results (45% of DCW) (Fig. 2c, Table 5) [29]. In addition, the ratio of unsaturated fatty acids to saturated fatty acids in strains YLpO and CYLpO was increased as a result of the conversion by SCD1 of palmitoyl-CoA and stearoyl-CoA substrates to their respective monounsaturated fatty acyl-CoAs (Fig. 2d).

**Table 5:** Comparison of lipid and biomass yield of recombinant *Y. lipolytica* strains grown on 80 g/L glucose or 72.8 g/L cellulose (C/N ratio ≈ 30:1) in shake flasks for 5 days

	*Y*_X/S_ (g DCW/g)	*Y*_FAME/S_ (g/g)	*Y*_FAME/X_ (g/g DCW)	*P*_FAME_ (g/L/h)
MM-glucose				
YLpW	0.26 ± 0.02	0.030 ± 0.004	0.12 ± 0.02	0.016 ± 0.002
YLpO	0.28 ± 0.01	0.092 ± 0.005	0.30 ± 0.02	0.073 ± 0.003
CYLpO	0.27 ± 0.02	0.088 ± 0.003	0.29 ± 0.01	0.070 ± 0.004
MM-cellulose^a^				
YLpO + 10 FPU/g	0.26 ± 0.02	0.058 ± 0.004	0.22 ± 0.01	0.024 ± 0.004
YLpO + 20 FPU/g	0.27 ± 0.00	0.091 ± 0.003	0.31 ± 0.03	0.073 ± 0.003
CYLpO	0.25 ± 0.03	0.036 ± 0.002	0.14 ± 0.02	0.005 ± 0.001
CYLpO + 10 FPU/g	0.26 ± 0.01	0.085 ± 0.004	0.30 ± 0.02	0.069 ± 0.002

Results were calculated from at least three biological replicates and are given as the mean values ± standard deviation

^a^Yields were calculated from the glucose consumption deduced from the cellulose degradation

**Fig. 3:**
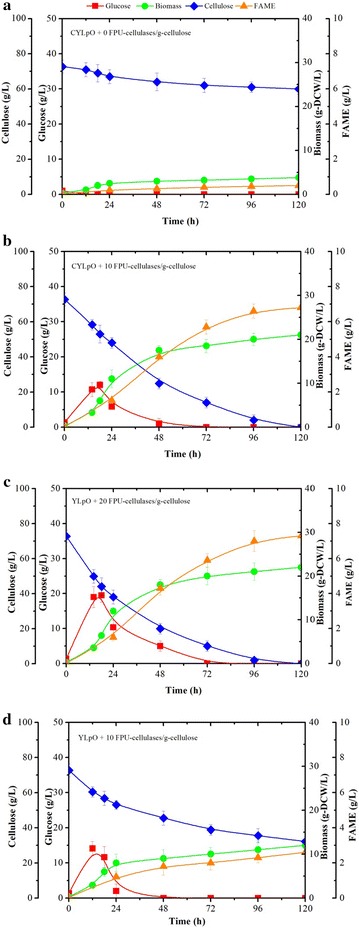
Comparison of growth, cellulose consumption and lipid production of recombinant *Y. lipolytica* strains. **a** CYLpO, **b** CYLpO with the addition of 10 FPU cellulases/g cellulose, **c** YLpO with the addition of 20 FPU cellulases/g cellulose and **d** YLpO with the addition of 10 FPU cellulases/g cellulose during aerobic batch culture in minimum media containing 72.8 g/L cellulose

We further studied the lipid production of the engineered strains with cellulose as carbon source instead of glucose. CYLpO consumed 12 g/L of cellulose and accumulated lipids up to 14% of its DCW (as measured by FAME), demonstrating that cellulolytic potency of CYLpO was insufficient to completely hydrolyze the cellulose, and consequently lipid production was limited by insufficient glucose availability (Fig. 3a). Since high carbohydrate to lipid conversion yields and high productivity will be essential to achieve the cost-effective production of biodiesel, we investigated how these parameters can be enhanced using CYLpO. For this, different loadings of Cellic^®^ CTec2 (5–30 U/g cellulose) were added to the culture medium to assist cellulose hydolysis. In the presence of an external cellulase loading of 20 FPU/g cellulose (Fig. 3c), YLpO was able to achieve rates of biomass and lipid production that are similar to those achieved by YLpO grown on glucose (Figs. 2, 3). Complete degradation of cellulose was achieved within less than 96 h and the final lipid concentration reached 31% of its DCW. Remarkably, in similar conditions CYLpO only required 10 FPU/g cellulose to maintain a performance equivalent to that when this strain is grown on glucose. Moreover, it is noteworthy that comparing the growth of CYLpO and YLpO in the presence of 10 FPU external cellulase/g cellulose revealed that yeast growth and lipid production in the latter strain were rather limited (Fig. 3b, d). This clearly highlights the added value of the endogenous cellulase production in CYLpO. Based on literature data regarding simultaneous saccharification and fermentation (SSF) processes, and the results presented herein, we estimate that the use of CYLpO would lead to a 50% reduction in the requirement for external cellulases in an SSF process. Additionally, it is important to note that the endogenous expression of cellulases by CYLpO did not alter the obese phenotype of the engineered strain when it was grown on glucose (i.e., the final fatty acid concentration for YLpO and CYLpO was essentially equivalent) (Figs. 2, 3, Table 4). Overall, these data reveal that cellulolytic CYLpO is an excellent candidate for the further development of a cellulose-based CBP for lipid production.

### Lipid production of recombinant *Y. lipolytica* strains in bioreactors

**Fig. 4:**
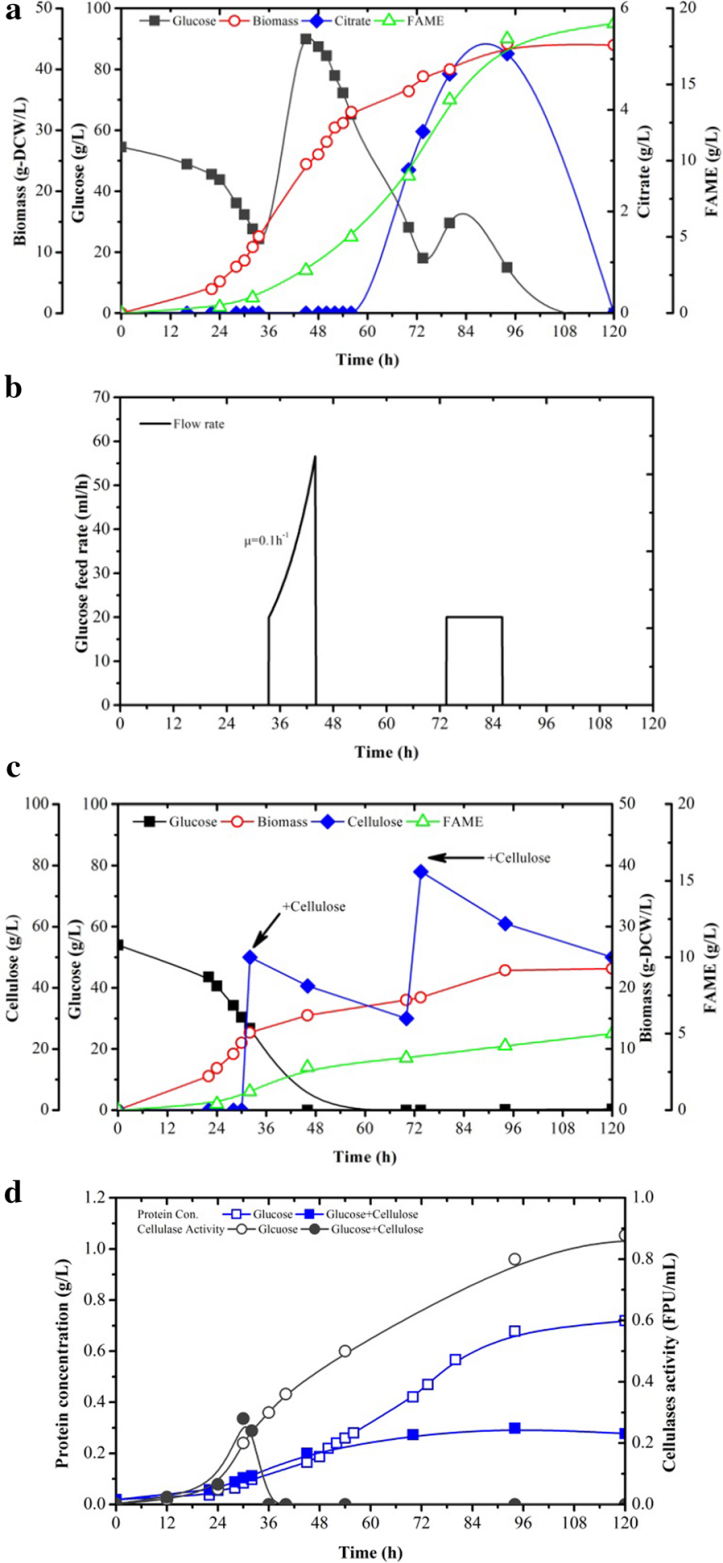
Comparison of growth, substrate consumption, cellulases secretion and lipid production of CYLpO in aerobic fed-batch cultures on glucose (**a**, **b** and **d**), and on glucose and cellulose (**c**, **d**) in bioreactors. Values plotted at each time point are the means of three replications. Error bars represent standard deviation from the means

To further explore the potential use of CYLpO as a CBP microorganism with autonomic feature, we evaluated the growth and lipid production of CYLpO in a 5.0-L bioreactor in minimal medium. Considering the recalcitrance of cellulose to cellulases degradation, CYLpO was first grown on glucose which allowed the strain to produce a certain amount of starter cellulases and then cellulose was supplemented into the bioreactors as carbon source. Fed-batch experiment with glucose feeding was used as control for comparison. During this control experiment, enhanced lipid biogenesis was observed for CYLpO, and a final FAME concentration of 19 g/L (43% of DCW) was achieved in 108 h (Fig. 4a, Table 6). A remarkable enhancement of biomass production was achieved by exponential glucose feeding (33–42 h) to a concentration that can be hardly reached in batch fermentation in shake flasks due to limited mixing (Fig. 4a, b). This accumulation of biomass observed for CYLpO is correlated with continuous increase in cellular fatty acid content. Indeed, 2.5-fold higher lipid productivity was achieved than that of shake flask fermentation, due to the fast establishment of a large amount of biomass (Tables 5 and 6).

**Table 6:** Comparison of lipid and biomass yield of recombinant *Y. lipolytica* grown on glucose and cellulose (C/N ratio ≈ 30:1) in aerobic fed-batch cultivations in bioreactors for 5 days

	*Y*_X/S_ (g DCW/g)	*Y*_FAME/S_ (g/g)	*Y*_FAME/X_ (g/g DCW)	*P*_FAME_ (g/L/h)
Glucose (200 g/L)	0.22	0.10	0.43	0.18
Glucose (50 g/L) + cellulose (50 g/L)^a^	0.22	0.05	0.22	0.04

^a^Yields were calculated from the consumption of glucose deduced from cellulose degradation

For fatty acid production on cellulose, CYLpO consumed 50 g/L of cellulose and produced 5.0 g/L FAME in 96 h (Fig. 4c). Continuous cellulase production was observed for CYLpO grown on glucose and this was accompanied by an increase in extracellular protein concentration in the culture medium (Fig. 4d). However, cellulase activity decreased greatly when cellulose was added to the culture medium and in the, meanwhile, the extracellular protein concentration remained low and constant, which indicated that most of the cellulases were absorbed onto the substrate. Our results also showed that further cultivation did not yield significant utilization of the residual cellulose after 120 h, which illustrated that the process was limited by the availability of sufficient free cellulase activity (Fig. 4d). The cellulose to lipid (measured as FAME) conversion yield was 0.05 g/g in cellulose feeding phase after glucose depletion, 50% of which was obtained on glucose (Table 6). If the entire process is taken into account, including cell growth on glucose, the lipid productivity was 0.04 g/L/h, although lower than the previously reported engineered *Y. lipolytica* strains [13, 29, 30]; further improvement of lipid production could be expected by overexpressing ACC1 in CYLpO, as shown previously [13]. In the future, further studies will focus on process optimization, either determining the exact glucose/cellulose ratio required to produce the highest cellulase titer during the early culture phase, or in the case of external cellulases, refining the enzyme loading to achieve maximum cellulose hydrolysis at the lowest enzyme cost. In addition, a continuous process will be developed to reuse the leftover cellulose, because this will already contain bound recombinant cellulases. In summary, our work has revealed the potentiality of the engineered cellulolytic *Y. lipolytica* for the bioconversion of cellulose to biodiesel ingredients.

### Lipase production using recombinant *Y. lipolytica* strains

Proteins are high value products of great interest for both research and commercial purposes. *Y. lipolytica* is an attractive host for protein production, because it exhibits remarkable ability to secrete a whole range of heterologous proteins [22]. Moreover, our previous studies aimed at conferring cellulolytic ability to *Y. lipolytica* have revealed that this yeast can simultaneously produce multiple proteins, secreting several of these into the culture medium. In this study, a native lipase (lipase 2), an enzyme that is widely used in wastewater treatment, chemical synthesis and pharmaceutical industries [21, 31], was overexpressed in CYLp, resulting in strain CYLpL. Using this strain, we have investigated how simultaneous cellulase production affects lipase production and how this strain can be used to drive cellulose-based recombinant protein production.

**Fig. 5:**
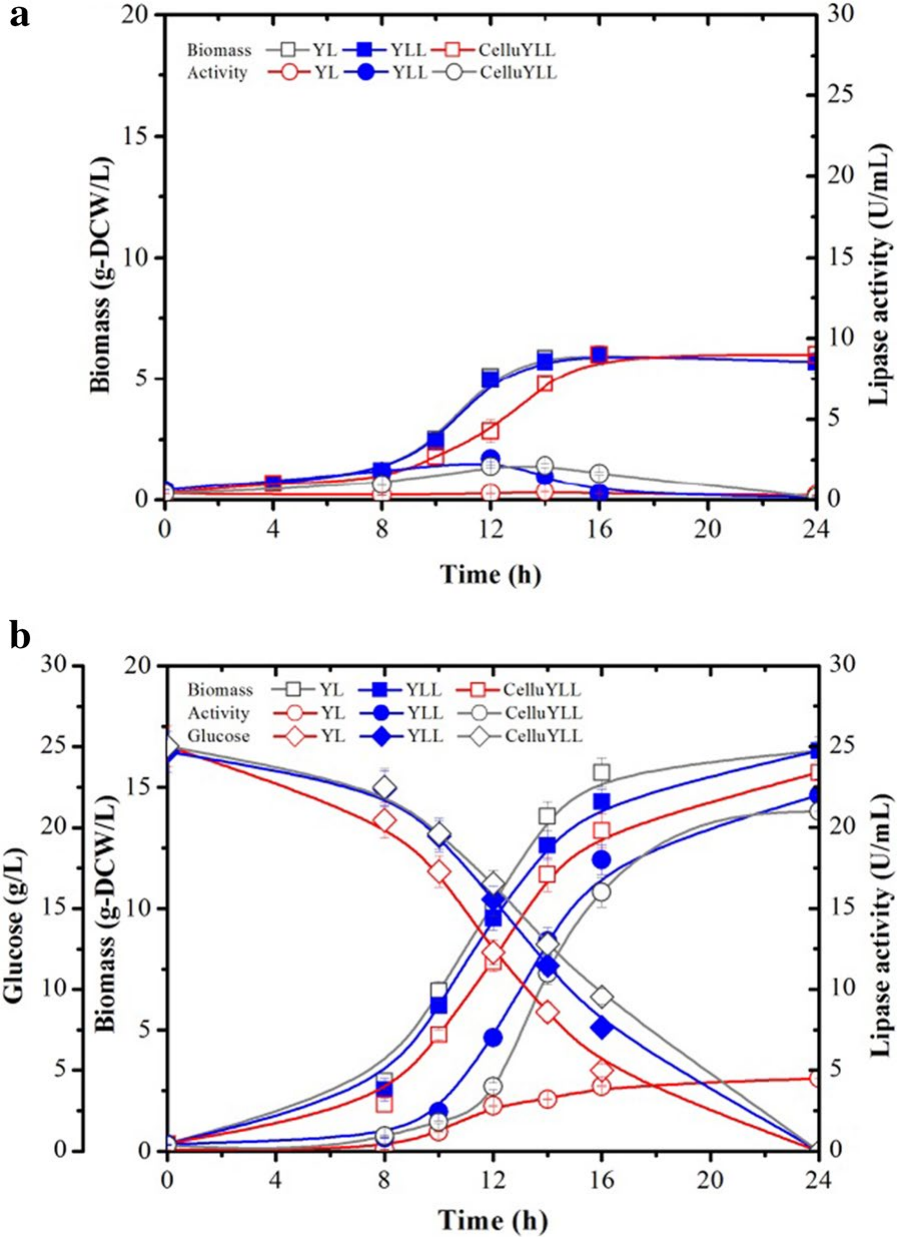
Comparison of the growth, sugar consumption and lipase production of recombinant *Y. lipolytica* strains YLpW, YLpL (∆pox strain overexpressing *LIP2*) and and CYLpL (cellulolytic ∆pox strain overexpressing *LIP2*) during aerobic batch cultures on **a** YT and **b** YTD media versus time. Values plotted at each time point are the means of three replications. Error bars represent standard deviation from the means

Both CYLpL and the control strains, YLpW and YLpL, were able to grow on YT medium (6.0 g DCW/L in 16 h) even when glucose was absent (Fig. 5a). However, recombinant strains YLpL and CYLpL grown on YTD produced five times higher lipase activity (22.0 U/mL in 24 h) than YLpW (Fig. 5). It is likely that the background activity of lipase for YLpW (4.5 U/mL) is indicative of the low level expression of the native lipases 2, 7 and 8, which are expressed at high level upon the induction with fatty acids [21] (Fig. 5). Significantly, lipase production did not affect growth rate, biomass yield, sugar consumption and cellulase production, since these were approximately the same for the strains tested when these were grown in YTD minimal medium (Table 4). Overall, the cellulolytic *Y. lipolytica* proved to be an efficient host for protein production, producing 800 U lipase/g glucose and, in parallel, multiple cellulases.

**Fig. 6:**
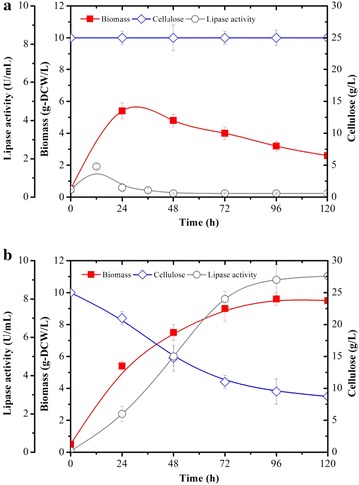
Comparison of the growth, cellulose consumption and lipase production of recombinant *Y. lipolytica* strains **a** YLpL and **b** CYLpL during aerobic cultures on YTC media versus time. Values plotted at each time point are the means of three replications. Error bars represent standard deviation from the means

Logically, attempts to perform cellulose-based lipase production on YTC using YLpL (Figs. 5a; 6a) led to the accumulation of only 3.0 g DCW/L, low lipase production (even after 120 h) and no cellulose consumption. Contrastingly, the use of CYLpL led to the consumption of 16 g/L of cellulose, which correlated with better growth (10 g DCW/L) and lipase production (9.0 U/mL after 96 h). In terms of lipase yield, CYLpL produced 562 U lipase/g cellulose. Unsurprisingly this is lower than the optimal yield (880 U lipase/g glucose) obtained using glucose, but the achievement is nonetheless remarkable, because to our knowledge this is the first time that a CBP approach has been used to produce recombinant proteins (other than cellulases) using cellulose as the carbon feedstock. Building on this proof of concept, it is easy to imagine how this platform could be used to produce a variety of valuable proteins or multifunctional peptides, for example for pharmaceutical applications. For future work, it might be necessary to improve the efficiency and/or productivity of the cellulolytic arsenal produced by CYLpL, either by replacing certain components with alternative enzymes, or using enzyme engineering techniques to improve the existing ones. However, prior to this it will be necessary to perform a detailed physiological study of the engineered strains to acquire systemic understanding of how engineering has impacted the metabolic pathways and regulation networks. This is vital to better satisfy energy requirements and anticipate competition for growth intermediates and protein production.

### Ricinoleic acid production of recombinant *Y. lipolytica* strains

The parental strain YLx, which is deprived of the native triacylglycerol (TAG) acyltransferases (Dga1p, Dga2p, and Lro1p) and the ∆12 desaturase (Fad2p), is a promising host for the specific production of unconventional fatty acid, such as ricinoleic acid (RA, C18:1 12-OH). This fatty acid and its derivatives are important oleo-chemicals which have numerous applications. A previous study has demonstrated that the expression of *CpFAH12* in YLx enabled RA production [22]. In the current study, we first endowed YLx with cellulolytic capability (i.e., creating CYLx) and then introduced *CpFAH12,* thus creating CYLxR, which is designed to allow CBP of cellulose to RA (Table 1). Secretion of RA into the culture medium was achieved by adding 10% of decane, which normally yielded more than 95% of RA recovery (data not shown).

**Fig. 7:**
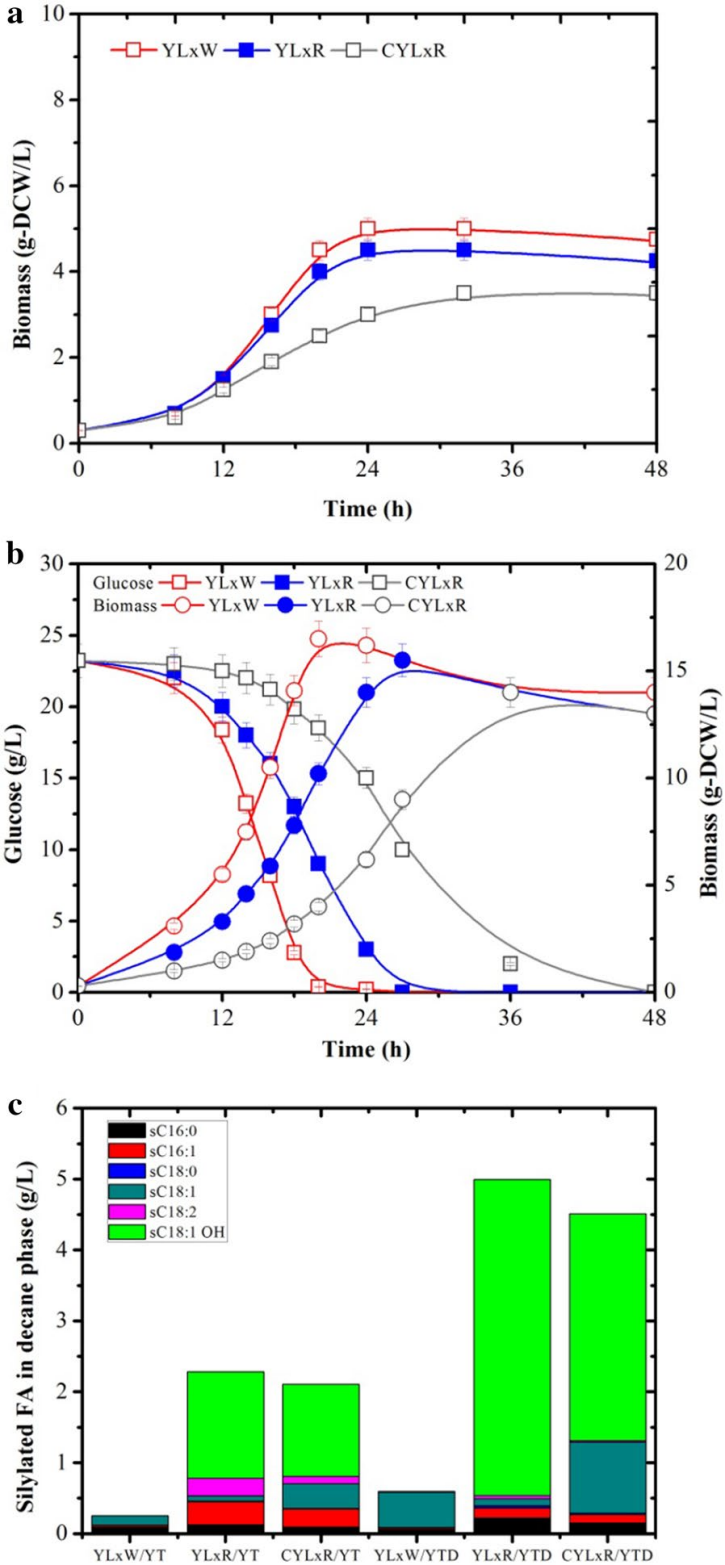
Comparison of growth and glucose consumption of recombinant *Y. lipolytica* strains YLxW (prototrophic OleoX strain), YLxR (OleoX strain overexpressing *CpFAH12*) and CYLxR (cellulolytic OleoX strain overexpressing *CpFAH12*) during aerobic batch culture in **a** YT media and **b** YTD media versus time, and **c** ricinoleic acid production in 2 days. Values plotted at each time point are the means of three replications. Error bars represent standard deviation from the means

**Fig. 8:**
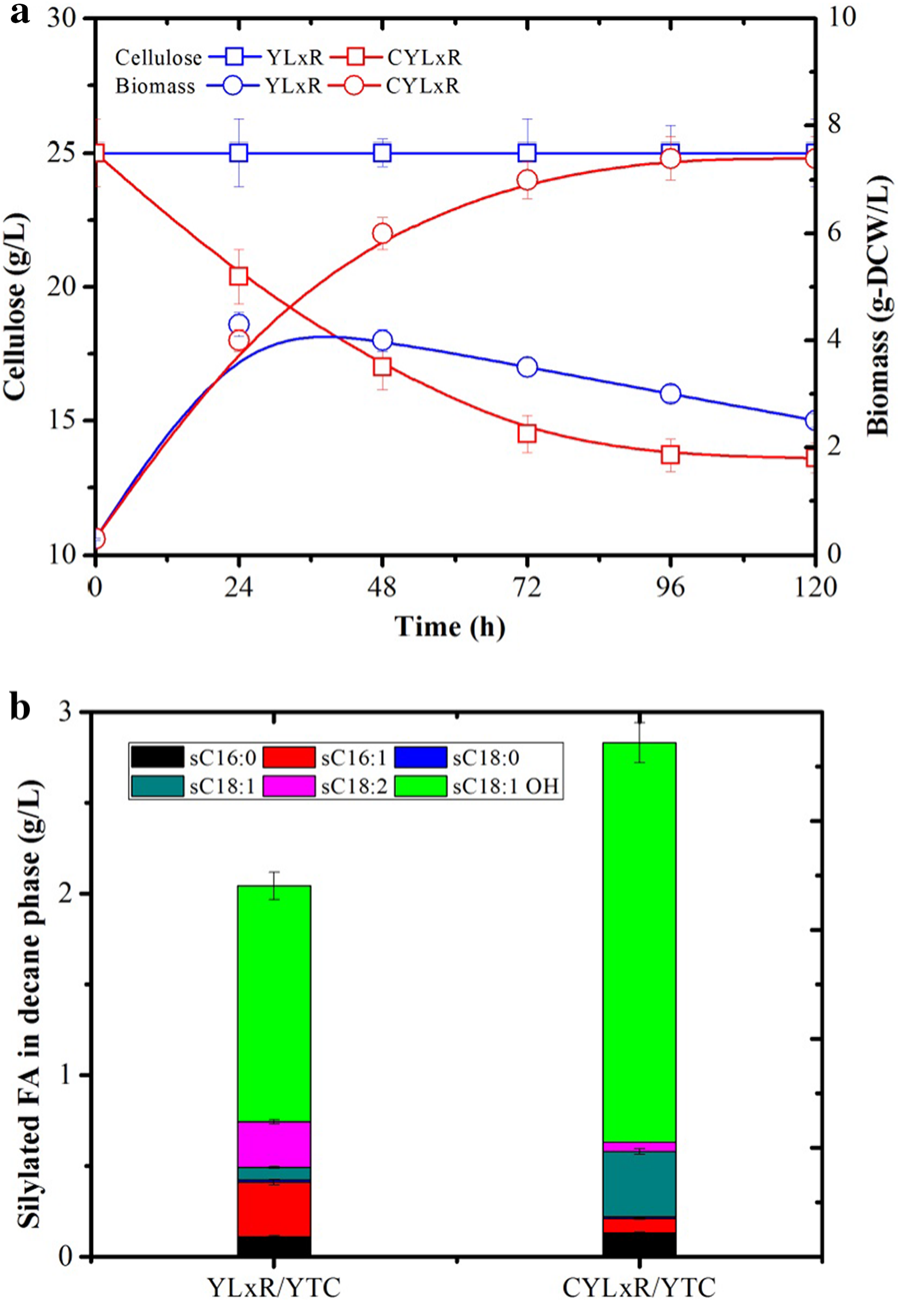
Ricinoleic acid production of recombinant *Y. lipolytica* strains YLxR and CYLxR during aerobic batch culture in YTC media. **a** Comparison of growth and cellulose consumption and **b** ricinoleic acid production in 5 days. Values plotted at each time point are the means of three replications. Error bars represent standard deviation from the means

Cultivation of the prototrophic YLxW strain, YLxR (expressing *CpFAH12*) and CYLxR in MM and YTD media revealed that the expression of *CpFAH12* led to a 27% decrease in *μ*_max_, but similar biomass yield and cellulolytic activity (for CYLxR) compared to the parental strain CYLx (Table 4). For all the cultures, no consumption of citrate was observed in 5 days of cultivations. Additionally, although YLxR and CYLxR showed lower growth rates than YLxW when grown on YT and YTD, probably due to the toxicity of RA accumulation [22], the final biomass yield of the three strains was essentially the same (Fig. 7). Consequently, CYLxR produced 1.3 and 3.2 g RA/L decane in 36 h on YT and YTD respectively, while YLxR produced higher amounts over a shorter period (1.5 and 4.4 g RA/L decane in 28 h, respectively), thus illustrating the effect of the cellulase production burden (Fig. 7c). However, the control strain YLxW did not produce RA on either media. When grown on YT containing cellulose as carbon source (YTC), CYLxR consumed 11 g/L cellulose and produced 7.0 g/L biomass and 2.2 g RA/L decane in 96 h, while YLxR produced maximum 4.0 g/L biomass and 1.3 g/L RA without the consumption of cellulose (Fig. 8). Moreover, the fatty acid profiles in the decane phases of CYLxR and YLxR were different, especially regarding the amount of oleic acid (C18:1), which was four to ten times higher in the case of CYLxR when grown on the different media (YT, YTD and YTC). However, the reason for the increased secretion of level of oleic acid in CYLxR remains to be elucidated. This higher level of oleic acid production (a RA precursor) by CYLxR might explain why RA production is lower than that in YLxR (Figs. 7c and 8b). The CYLxR-mediated production yield of RA on cellulose (0.18 g RA/g glucose, calculated from the cellulose consumed) was similar to that of YLxR grown on glucose (0.19 g RA/g glucose), which was close to the highest reported RA yield so far [22]. However, the productivity of RA on cellulose was about 0.02 g/L/h, which only represents 25.8% of that obtained on glucose (0.09 g/L/h). Since RA production is growth dependent, the low RA productivity of CYLxR can be ascribed both to the low robustness of the YLx background strain and the insufficient cellulolytic activity of CYLxR, which both combine to limit growth on cellulose. Robustness is a vital attribute for strains that are used in lignocellulosic biorefineries, since the pretreated biomass hydrolysates create challenging process conditions. In future work, further engineering of YLx derivatives will be required to boast strain robustness.

## Conclusion

Herein, we describe the further engineering of a cellulolytic *Y. lipolytica* strain aimed at optimizing cellulase production and conferring the ability to produce a number of target products. Accordingly, we have exemplified the potential of this strain for use as a CBP host for the production of recombinant proteins, lipids and ricinoleic acid. Shortcomings, such as the suboptimal rate of cellulose hydrolysis, have been highlighted as remaining challenges that will require further work. Improvement of the cellulase arsenal will probably require both enzyme engineering and the recruitment of other “accessory” enzymes. However, a prerequisite for further strain engineering is a systems-level analysis aimed at carefully identifying and studying metabolic bottlenecks and rate-limiting steps. Moreover, in addition to further strain improvement, economic analysis of hybrid process scenarios including a continuous process might also be worthwhile to determine whether it is feasible to reinforce the inherent cellulolytic capability of engineered *Y. lipolytica* by the addition of external cellulases. Overall, the engineered cellulolytic *Y. lipolytica* strain described herein can be described as a promising prototype for the development of CBP aimed at converting cellulose into a wide variety of commercially relevant products.

## Additional file

**Additional file 1: Figure S1.** Schematic diagram of the strain constructions. **Figure S2.** Schematic diagram of the construction of the plasmid JMP62LeuEB1TE1 for co-expressing BGL1 and EG1. **Figure S3.** Schematic diagram of the construction of the plasmid JMP62UraTB2EE2 for co-expressing BGL2 and EG2. **Figure S4.** PCR verification of *Y. lipolytica* transformants expressing multiple cellulases and genes for producing target products (A) CYLpL, Lane 1 to 7: *YlBGL1, YlBGL2, TrEG1, TrEG2, NcCBH1, TrCBH2, YlLIP2*; (B) CYLpO, Lane 1 to 8: *YlBGL1, YlBGL2, TrEG1, TrEG2, NcCBH1, TrCBH2, YlSCD1, YlDGA1*; (C) CYLxR, Lane 1 to 7: *YlBGL1, YlBGL2, TrEG1, TrEG2, NcCBH1, TrCBH2, CpFAH12*. **Figure S5.** The schematic diagram of the strain construction strategies (A) the previous strategy which easily caused gene loss; (B) the current strategy to avoid gene loss by reducing *LoxP* sites. **Figure S6.** Comparison the production of (a) BGL1 and (b) EG2 under the control of EXP and TEF promoter by *Y. lipolytica* grown on YTD media. **Figure S7.** Phase contrast and fluorescence microscopy of intracellular stored lipids stained with Bodipy dye of the recombinant strains during aerobic batch culture in minimal media supplemented with glucose or cellulose. (a, b) YLpW, (c, d) YLpO and (e, f) and CYLpO on glucose; (g, h) CYLpO on cellulose with supplementation of cellulases at 10 FPU/g cellulose, (i, j) YLpO on cellulose with supplementation cellulases at 20 and (k, l) 10 FPU/g cellulose. **Table S1.** The sequences of the oligonucleotide primers used for PCR verification of *Y. lipolytica*-transformants.

## Abbreviations

ACC1: acetyl-CoA carboxylase
BGL: β-glucosidase
CBH: cellobiohydrolase
CBP: consolidated bioprocessing
CMC: carboxymethyl cellulose
*Cp*FAH12: *Claviceps purpurea* fatty acid Δ12-hydroxylase
DCW: dry cell weight
DGA1: acyl-CoA:diacylglycerol acyltransferase 1
EG: endoglucanase
FAMEs: fatty acid methyl esters
FPU: filter paper units
LCB: lignocellulosic biomass
MM: minimal medium
NREL: National Renewable Energy Laboratory
*P*_FAME_: productivity of FAMEs
pNPB: *p*-nitrophenyl butyrate
RA: ricinoleic acid
SCD1: stearoyl-CoA desaturase
SSF: simultaneous saccharification fermentation
YL: *Y. lipolytica*
