# Supplementary material for: Developing cellulolytic Yarrowia lipolytica as a platform for the production of valuable products in consolidated bioprocessing of cellulose
Source: Biotechnol Biofuels. 2018 May 15;11:141. doi: 10.1186/s13068-018-1144-6 (PMC5952637; doi:10.1186/s13068-018-1144-6)
Supplement: Supplementary file 1 — Additional file 1: Figure S1. Schematic diagram of the strain constructions. Figure S2. Schematic diagram of the construction of the plasmid JMP62LeuEB1TE1 for co-expressing BGL1 and EG1. Figure S3. Schematic diagram of the construction of the plasmid JMP62UraTB2EE2 for co-expressing BGL2 and EG2. Figure S4. PCR verification of Y. lipolytica transformants expressing multiple cellulases and genes for producing target products (A) CYLpL, Lane 1 to 7: YlBGL1, YlBGL2, TrEG1, TrEG2, NcCBH1, TrCBH2, YlLIP2; (B) CYLpO, Lane 1 to 8: YlBGL1, YlBGL2, TrEG1, TrEG2, NcCBH1, TrCBH2, YlSCD1, YlDGA1; (C) CYLxR, Lane 1 to 7: YlBGL1, YlBGL2, TrEG1, TrEG2, NcCBH1, TrCBH2, CpFAH12. Figure S5. The schematic diagram of the strain construction strategies (A) the previous strategy which easily caused gene loss; (B) the current strategy to avoid gene loss by reducing LoxP sites. Figure S6. Comparison the production of (a) BGL1 and (b) EG2 under the control of EXP and TEF promoter by Y. lipolytica grown on YTD media. Figure S7. Phase contrast and fluorescence microscopy of intracellular stored lipids stained with Bodipy dye of the recombinant strains during aerobic batch culture in minimal media supplemented with glucose or cellulose. (a, b) YLpW, (c, d) YLpO and (e, f) and CYLpO on glucose; (g, h) CYLpO on cellulose with supplementation of cellulases at 10 FPU/g cellulose, (i, j) YLpO on cellulose with supplementation cellulases at 20 and (k, l) 10 FPU/g cellulose. Table S1. The sequences of the oligonucleotide primers used for PCR verification of Y. lipolytica-transformants. [file 13068_2018_1144_MOESM1_ESM.pdf]

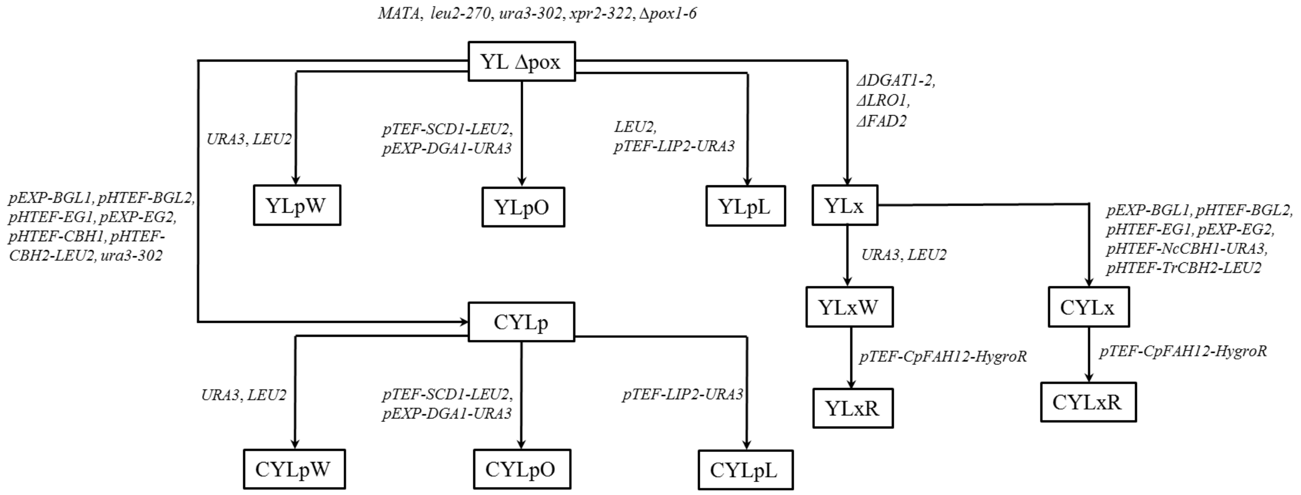

Figure S1 Schematic diagram of the strain constructions.

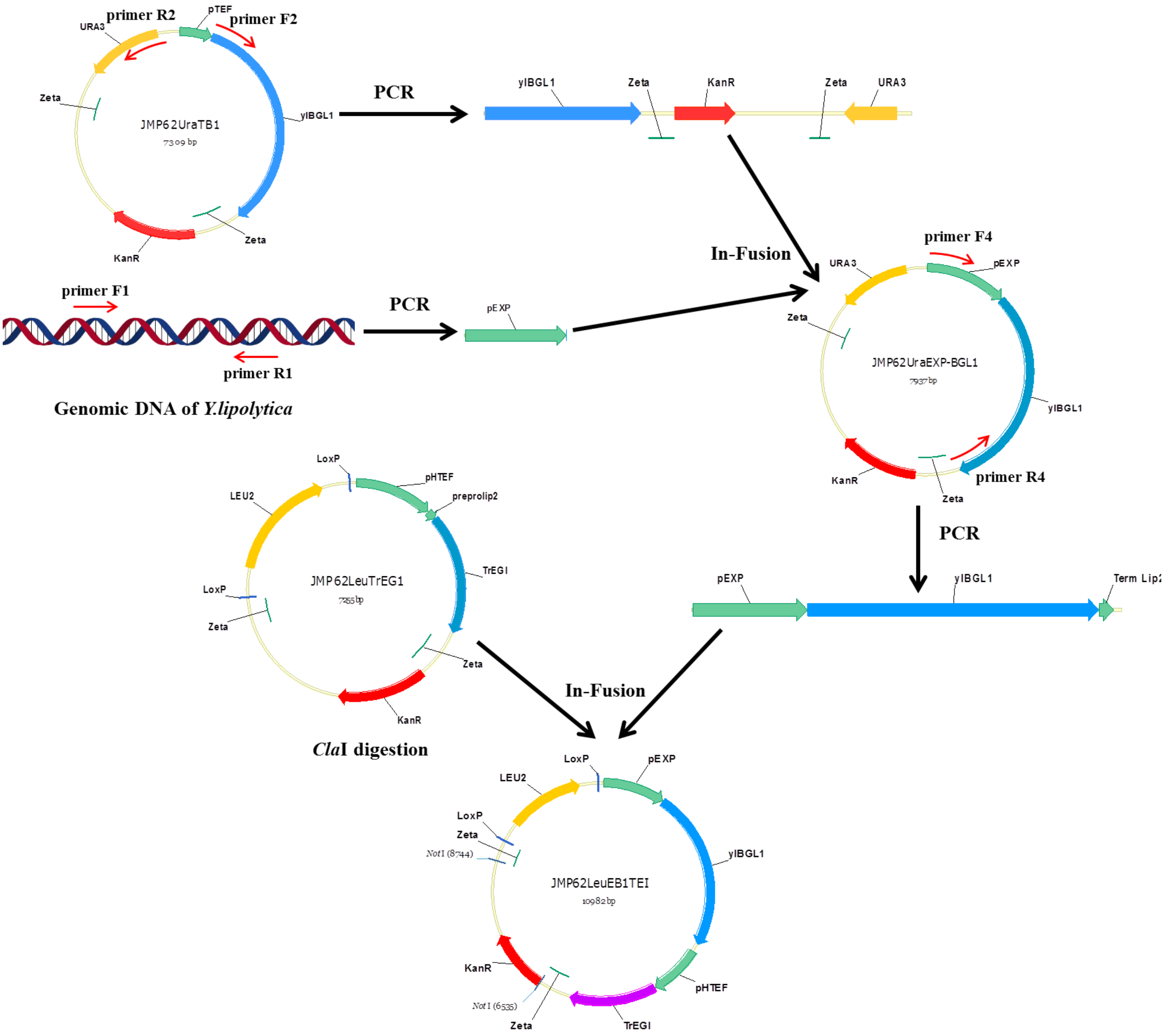

Figure S2 Schematic diagram of the construction of the plasmid JMP62LeuEB1TE1 for co-expressing BGL1 and EG1.

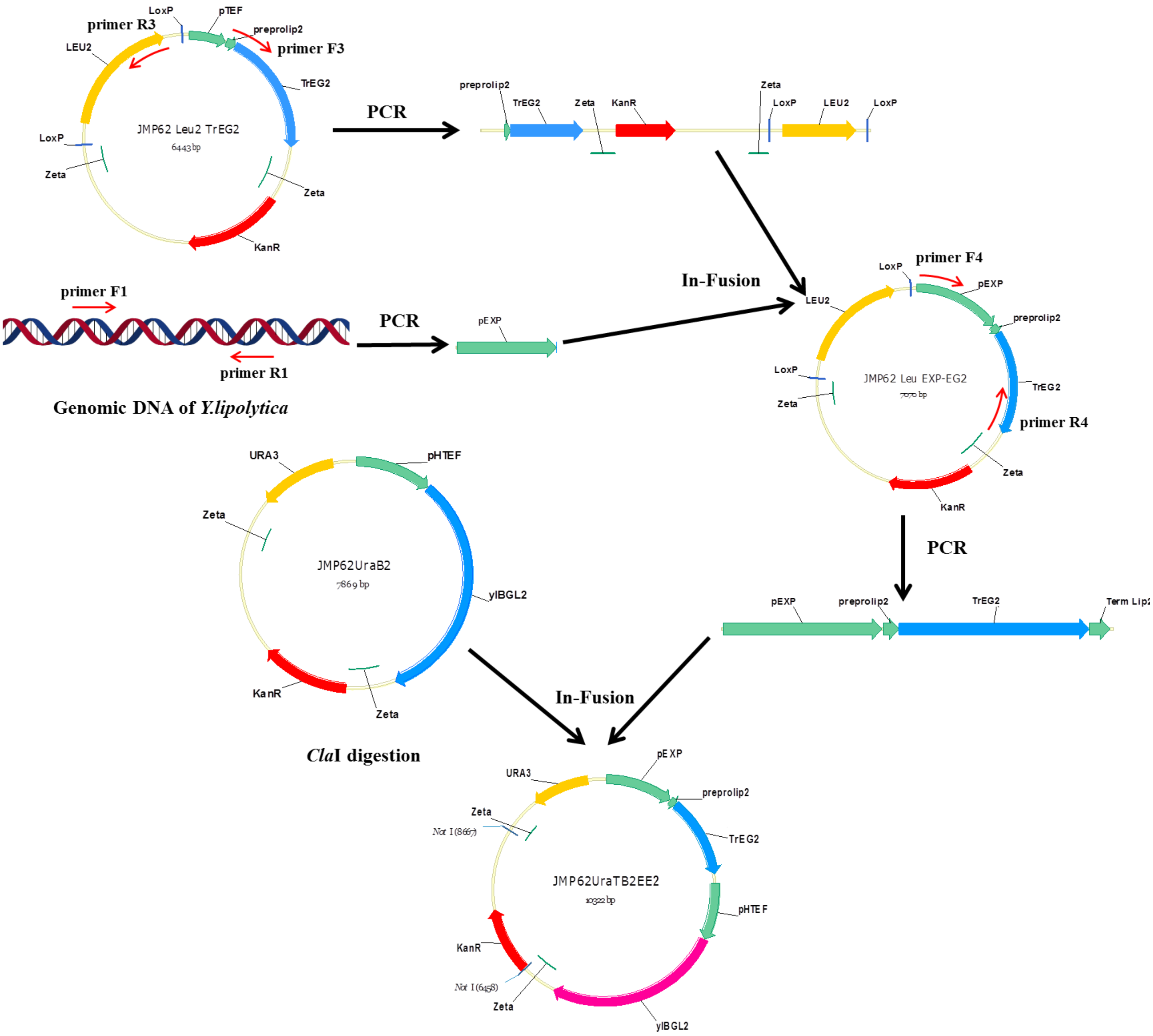

Figure S3 Schematic diagram of the construction of the plasmid JMP62UraTB2EE2 for co-expressing BGL2 and EG2.

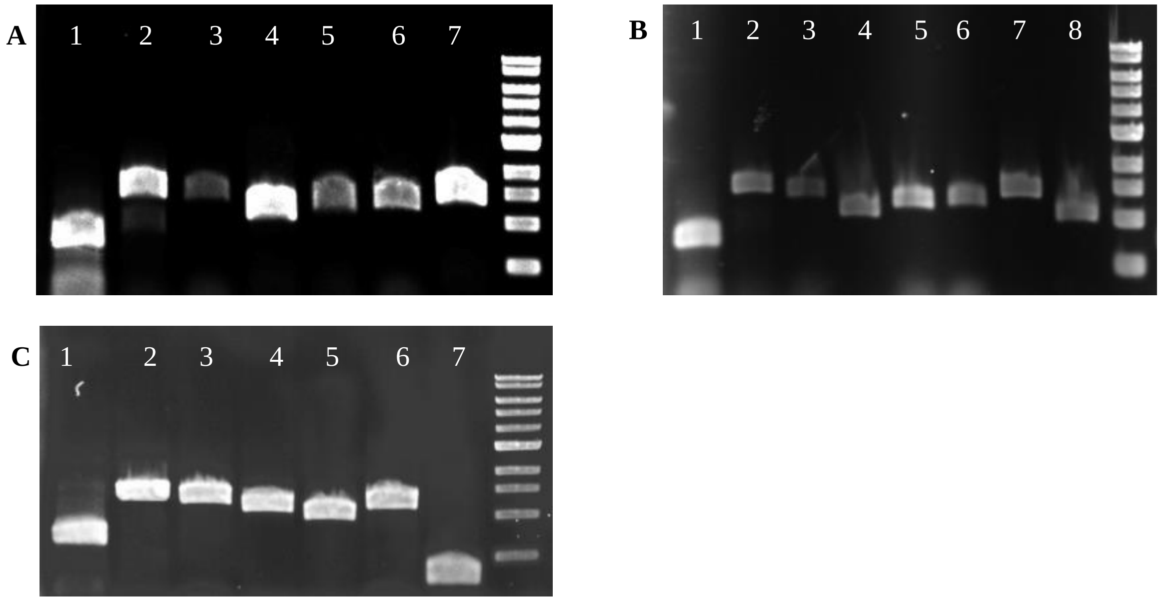

Figure S4 PCR verification of *Y. lipolytica* transformants expressing multiple cellulases and genes for producing target products (A) CYLpL, Lane 1 to 7: *YlBGL1*, *YlBGL2*, *TrEG1*, *TrEG2*, *NcCBH1*, *TrCBH2*, *YlLIP2*; (B) CYLpO, Lane 1 to 8: *YlBGL1*, *YlBGL2*, *TrEG1*, *TrEG2*, *NcCBH1*, *TrCBH2*, *YlSCD1*, *YlDGA1*; (C) CYLxR, Lane 1 to 7: *YlBGL1*, *YlBGL2*, *TrEG1*, *TrEG2*, *NcCBH1*, *TrCBH2*, *CpFAH12*.

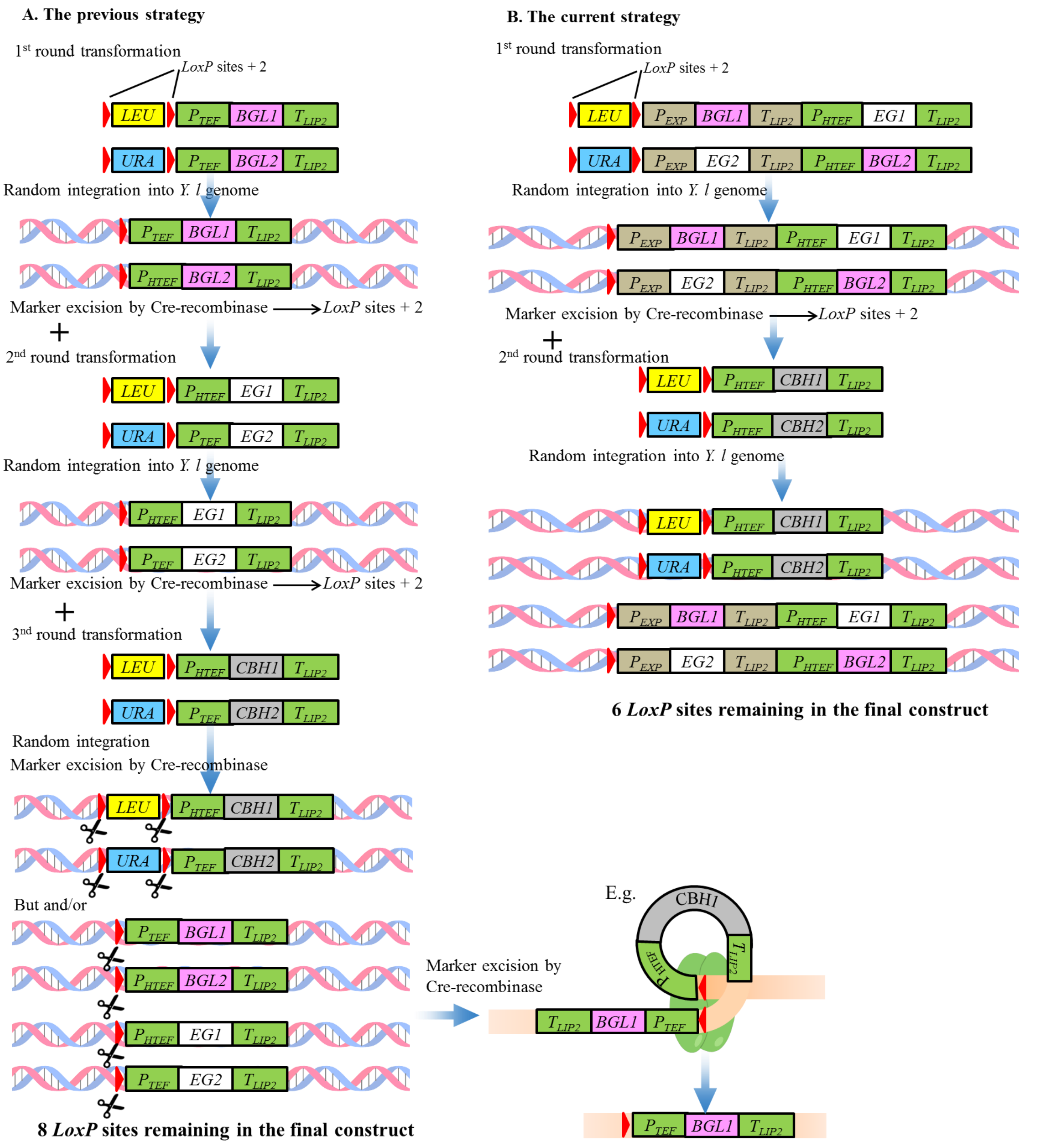

Figure S5 The schematic diagram of the strain construction strategies (A) the previous strategy which easily caused gene loss; (B) the current strategy to avoid gene loss by reducing *LoxP* sites.

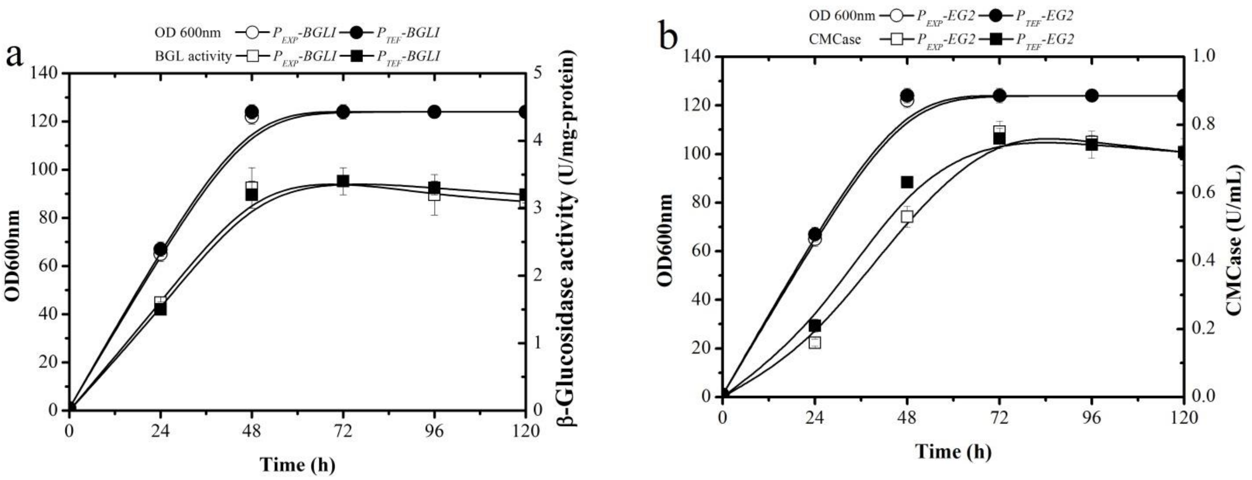

Figure S6 Comparison the production of (a) BGL1 and (b) EG2 under the control of EXP and TEF promoter by *Y. lipolytica* grown on YTD media.

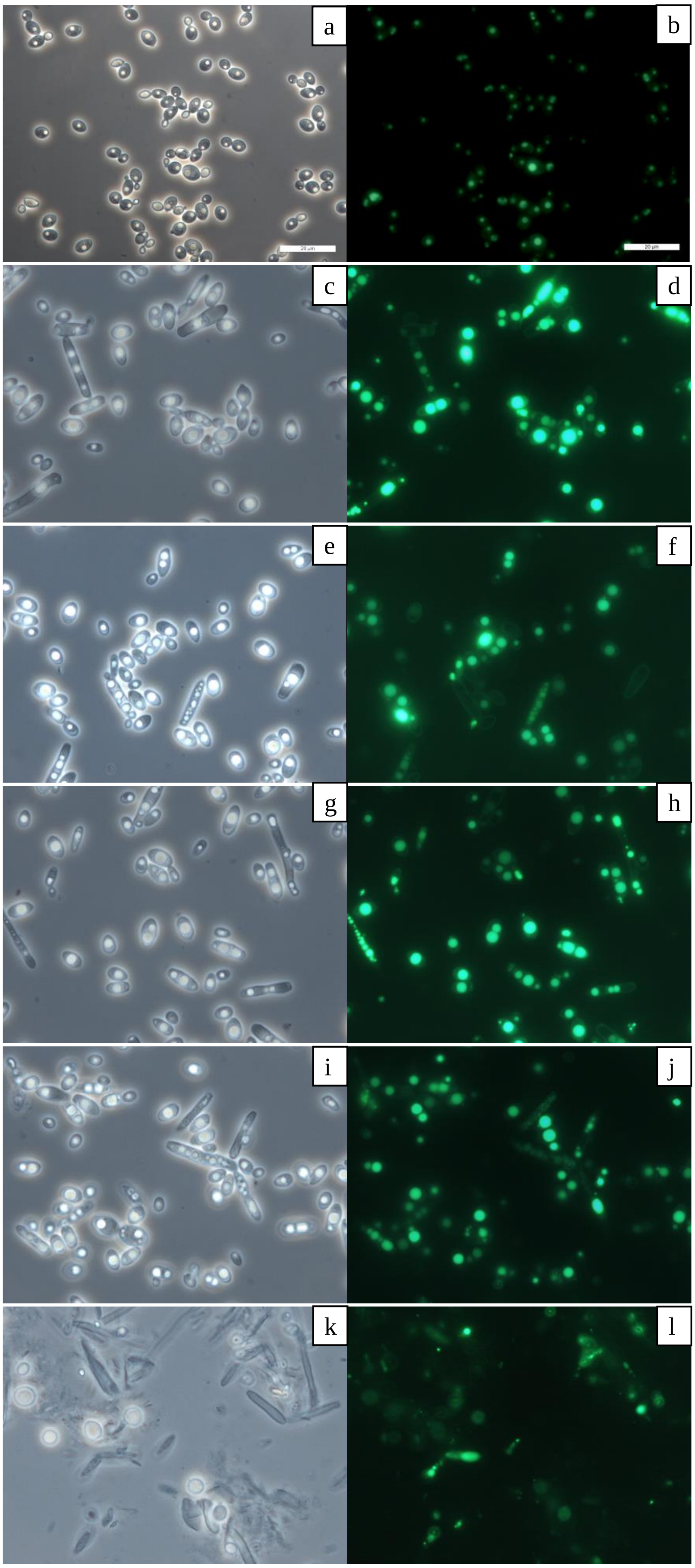

Figure S7 Phase contrast and fluorescence microscopy of intracellular stored lipids stained with Bodipy dye of the recombinant strains during aerobic batch culture in minimal media supplemented with glucose or cellulose. (a, b)

YLpW, (c, d) YLpO and (e, f) and CYLpO on glucose; (g, h) CYLpO on cellulose with supplementation of cellulases at 10 FPU/g-cellulose, (i, j) YLpO on cellulose with supplementation cellulases at 20 and (k, l) 10 FPU/g-cellulose.

Table S1 The sequences of the oligonucleotide primers used for PCR verification of *Y. lipolytica*-transformants

Primer names	Sequence (5’-3’)
VEXP-BGL1F	CCGTGACAGACGATGGGTGGCT
VEXP-BGL1R	GGTGAACTAGGATACGATCAAAGGTGGC
VTEF-BGL2F	GGTTGGCGGCGCATTTGT
VTEF-BGL2R	TGTCGTCCACTCGGCTTTCATC
V4UASTEF-EGIF	CACTTGCCGTTAAGGGCGTAGGGT
V4UASTEF-EGIR	CTGGCTGTTGTCGTTCCAAATGCTG
VEXP-EGIIF	GCTCGCATTGTCTGCCTTGTTTAGTTTG
VEXP-EGIIR	CGTAGCACCAGCGTTGCGGATT
V4UASTEF-CBHIF	CGCCGCAAGGAATGGTGCA
V4UASTEF-CBHIR	GGTTCGAGATCCGATGTTAGTGGAGTA
V4UASTEF-CBHIIF	CGCCGCAAGGAATGGTGCA
V4UASTEF-CBHIIR	CCGATCCGACTGGAGGTACTCTGGTAG
VTEF-LIP2F	ACCGGGTTGGCGGCGCATTT
YTEF-LIP2R	CCACAGACACCCTCGGTGACGAAGTA
VEXP-DGA1F	TGGGATTGTATGGAGTGGCATGGAGC
VEXP-DGA1R	TGAGGGACCCGTTAAGAAGCGTGGA
VTEF-SCD1F	ACACTTGCCGTTAAGGGCGTAGGG
YTEF-SCD1R	CGTCGTGGACAATGCCAGAAATGAG
VTEF-FAH12F	AGACCGGGTTGGCGGCGCATTT
YTEF-FAH12R	TGTCGCAGCACGGCGTTCTCAGACAT
